# Using wearable electronic sensors for assessing contacts between individuals in various environments

**DOI:** 10.1186/1753-6561-5-S6-O51

**Published:** 2011-06-29

**Authors:** N Voirin, J Stehlé, A Barrat, C Cattuto, L Isella, J-F Pinton, M Quaggiotto, N Khanafer, W Van den Broeck, C Régis, B Lina, P Vanhems

**Affiliations:** 1 Hospices Civils de Lyon - Université Lyon 1 - CNRS, Lyon, France; 2 CNRS - ISI Foundation, Marseille, France; 3 ISI Foundation, Torino, Italy; 4 ENS - CNRS, Lyon, France

## Introduction / objectives

Transmission of hospital acquired infections (HAI) is mainly based on contacts between patients, patients and health care workers (HCWs) and between HCWs. Description and quantification of contacts at hospital are key information for HAIs epidemiology and implementing control measures.

## Methods

The SocioPatterns project (http://www.sociopatterns.org) has developed an technology based on RFID badges that provides a reliable infrastructure to detect face-to-face proximity of individuals. The system was tested at a scientific conference, in a primary school and in a hospital unit.

## Results

At the scientific conference, 26,040 contacts (average duration of 54 seconds) were recorded among 402 participants during two days. At school, 77,226 contacts (average number of 160 contacts per children per day) were observed among 233 children during two days. In the geriatric unit, 50 staff and 29 patients participated to the study during 5 consecutive days. Statistical analyses are ongoing. For each study, time-resolved datasets on contact patterns were generated. Analyses provided frequency and duration of contacts, and contact matrices. Modeling of the spread of infections will be presented.

## Conclusion

The study of contacts networks is important to better address the prevention and control of known and emerging HAIs. They are also useful tools to build better models for decision making in public health. Collecting contacts data in the hospital setting, using such electronic devices, appeared to be appropriate and will be an added value to other approaches such as observational audit.

## Disclosure of interest

None declared.

